# Responses of Forest Net Primary Productivity to Climatic Factors in China during 1982–2015

**DOI:** 10.3390/plants11212932

**Published:** 2022-10-31

**Authors:** Ziqiang Du, Xuejia Liu, Zhitao Wu, Hong Zhang, Jie Zhao

**Affiliations:** 1 Institute of Loess Plateau, Shanxi University, Taiyuan 030006, China; 2 Shanxi Academy of Eco-Environmental Planning and Technology, Taiyuan 030000, China; 3 College of Environmental & Resource Science, Shanxi University, Taiyuan 030006, China; 4 College of Natural Resources & Environment, Northwest A & F University, Xianyang 712100, China

**Keywords:** forest ecology, net primary productivity, varying response, climate change, China

## Abstract

Forest ecosystems play an important role in the global carbon cycle. Clarifying the large-scale dynamics of net primary productivity (NPP) and its correlation with climatic factors is essential for national forest ecology and management. Hence, this study aimed to explore the effects of major climatic factors on the Carnegie–Ames–Stanford Approach (CASA) model-estimated NPP of the entire forest and all its corresponding vegetation types in China from 1982 to 2015. The spatiotemporal patterns of interannual variability of forest NPP were illustrated using linear regression and geographic information system (GIS) spatial analysis. The correlations between forest NPP and climatic factors were evaluated using partial correlation analysis and sliding correlation analysis. We found that over thirty years, the average annual NPP of the forests was 887 × 10^12^ g C/a, and the average annual NPP per unit area was 650.73 g C/m^2^/a. The interannual NPP of the entire forest and all its corresponding vegetation types significantly increased (*p* < 0.01). The increase in the NPP of evergreen broad-leaved forests was markedly substantial among forest types. From the spatial perspective, the NPP of the entire forest vegetation gradually increased from northwest to southeast. Over the years, the proportions of the entire forest and all its corresponding vegetation types with a considerable increase in NPP were higher than those with a significant decrease, indicating, generally, improvements in forest NPP. We also found climatic factors variably affected the NPP of forests over time considering that the rise in temperature and solar radiation improved the interannual forest NPP, and the decline in precipitation diminished the forest NPP. Such varying strength of the relationship between the interannual forest NPP and climatic factors also varied across many forest types. Understanding the spatiotemporal pattern of forest NPP and its varying responses to climatic change will improve our knowledge to manage forest ecosystems and maintain their sustainability under a changing environment.

## 1. Introduction

As an essential part of terrestrial ecosystems, vegetation is a natural link between the atmosphere, soil, and water. It not only plays an important role in the global material and energy cycles but also reduces the concentration of greenhouse gases. Net primary productivity (NPP) refers to the rate at which vegetation fixes carbon dioxide from the atmosphere in the ecosystem through photosynthesis minus the rate at which vegetation returns the carbon dioxide to the atmosphere through respiration. NPP stands for the net carbon input from the atmosphere into vegetation. It, therefore, is an important measure of ecosystem carbon sinks, carbon sources, and global carbon balance [1,2]. Increases in temperature, in the frequency, intensity, and distribution of precipitation, and in the spatiotemporal distribution of solar radiation will inevitably have the most important impact on the development, formation, and evolution of terrestrial ecosystems. Changes in NPP can reflect the responses of ecosystems to climatic conditions, so it can serve as an indicator of the responses of ecosystem functions to climate change.

As a dominant vegetation type around most of the world, forests comprise the major component of terrestrial ecosystems [3] and cover 3999 M ha globally in 2015 [4]. They account for about 80% of terrestrial carbon storage on the ground and 40% of it underground [5,6]. Forests can retain existing carbon stocks and effectively increase carbon sinks [7,8]. China’s forests represent a significant biomass carbon sink over the past several decades [9]. Because of the vast land area and heterogeneous natural conditions in China, its flora and forest types have diversified. The country harbors nearly all the types of forests in the northern hemisphere [10], ranging from boreal coniferous forests and broad-leaved forests, temperate deciduous broad-leaved forests, warm temperate or subtropical evergreen broad-leaved forests to tropical rainforests [11,12]. Its diverse climatic conditions and forest resources provide a good experimental base for studying the dynamics of the relationship between forest vegetation productivity and climate change [10,13].

Forest NPP is a key parameter that characterizes the functions of forest ecosystems, and it can be estimated to assess the development of forest ecosystems. The large-scale variations in forest NPP and its responses to climatic factors are increasingly gaining concern [2,10,14]. Large scale forest NPP estimates are of increasing interest. For example, Fang et al. [15] explored the spatial pattern of forest NPP in different provinces of China in terms of the third forest survey data. Zhou et al. [10] calculated China’s Larix forest NPP based on forest inventory data. Wang et al. [16] measured the NPP in different forests at site level in northern China with the boreal ecosystem productivity simulator. Hasenauer et al. [17] estimated the Australian forest productivity by reconciling satellite with ground data. Cao et al. [14] estimated aboveground NPP in secondary tropical dry forests at the Santa Rosa National Park, Costa Rica using the Carnegie–Ames–Stanford Approach (CASA) model. Tripathi et al. [18] estimated NPP in tropical forest plantations of India during 2009 and 2010 using CASA model. NPP is influenced by different factors, especially climate variables, as the main factors that affect the development and ranges of forest ecosystems. For instance, Peng et al. [19] reported that climate change led to an increase in the NPP of boreal forests in central Canada. Schuur and Matson [20] found that the underground NPP decreased 2.2 times with the increase in average annual precipitation in the mountain forests of Hawaii. Mohamed et al.. [21] stated that the variability in NPP of global ecosystems particularly forests and grasslands was attributed to global anomalies in temperature, precipitation and cloud cover. Cleveland et al. [22] noted that the mean annual temperature could be the strongest predictor of the ground NPP of all tropical forests, but this relationship may be caused by the apparent temperature difference between highland and lowland forests. Reyer et al. [23] suggested that future forest productivity would be subject to climate change, which in turn would largely depend on climate scenarios and the sustainability of CO_2_ impacts. Yao et al. [9] quantified Chinese forest biomass carbon sequestration capacity in the near future integrating the effects of stand development, climate change, and increasing CO_2_ concentration. Although previous studies have focused mainly on either forest NPP or the impact of climatic factors on forest NPP, most of them were limited at specific regions or within shorter periods [24]. Moreover, previous researchers have paid relatively little attention to the varying responses of vegetation growth (e.g., forest NPP) to climate variables [25].

We aimed to investigate the spatiotemporal variations in China’s forest NPP and explored the dynamic responses of forests in terms of their NPP to major climatic factors from 1982 to 2015, which were important for sustainable forest ecology and management.

## 2. Data and Methods

### 2.1. Data Sources

#### 2.1.1. Remote Sensing Data

We utilized the Global Inventory Modeling and Mapping Studies (GIMMS) Normalized Difference Vegetation Index-3rd generation (NDVI3g) remote sensing data generated by the National Oceanic and Atmospheric Administration (NOAA) Advanced Very High-resolution Radiometer (AVHRR) and provided by the National Aeronautics and Space Administration (NASA). This data set from 1982 to 2015, with 15-day time resolution and 0.083° spatial resolution, is the longest sequence of NDVI data and has been widely used in the estimation and investigation of large-scale vegetation dynamics, vegetation NPP, and biomass [26]. The effects of cloud and atmospheric interferences were eliminated and the monthly NDVI data were processed through the maximum-value composite (MVC) method [27].

#### 2.1.2. Meteorological Data

The monthly data, such as total solar radiation, average temperature, and precipitation over the same period as the remote sensing data, were obtained from the records of meteorological stations (Figure S1) in the China Meteorological Science Data Sharing Service System (CMSDS). Spatial interpolation was performed through inverse distance weighting (IDW) to generate meteorological grid data with spatial resolution and projection similar to that of the NDVI data [28,29].

#### 2.1.3. Forest-Type Data

Forest data were extracted from China’s 1:1,000,000 vegetation map provided by the Environmental and Ecological Science Data Center for West China, National Natural Science Foundation of China (http://westdc.westgis.ac.cn (accessed on 9 October 2022)). Forest vegetation types (Figure S1) included evergreen broad-leaved forest, deciduous broad-leaved forest, evergreen coniferous forest, deciduous coniferous forest, and mixed forest [30]. The distribution of evergreen broad-leaved forests is mostly in subtropical regions; deciduous broad-leaved forests are in temperate regions; evergreen coniferous forests are mostly south of the Qinling–Huaihe line; deciduous coniferous forests are in Northeastern and Northwestern China; and mixed forests are mostly in the Changbai Mountains, Xiaoxinganling Mountains, and subtropical mountains.

### 2.2. Methods

#### 2.2.1. CASA Model

NPP prediction models have become powerful alternative tools for investigating the NPP scale and geographic distribution of vegetation because the direct measurement of terrestrial vegetation NPP at the regional or global scale has been difficult [31]. The Carnegie–Ames–Stanford Approach (CASA) model is an NPP simulation model considered more realistic than others [1,14,32]. We used it in this study to estimate the NPP of the entire forest and the forest vegetation types and calculated it as follows:

(1) NPP(x,t)=APAR(x,t)×ε(x,t)

where *APAR*(*x*, *t*) represents the photosynthetically active radiation (PAR, in units of MJ/m^2^) absorbed at pixel *x* in month *t*, and *ε*(*x*, *t*) represents the actual light energy utilization at pixel *x* in month *t* (g C/MJ). APAR was computed as follows:

(2) APAR(x,t)=SOL(x,t)×FPAR(x,t)×0.5

where *SOL*(*x*, *t*) represents the total solar radiation (MJ/m^2^) at pixel *x* in month *t*; *FPAR*(*x*, *t*) represents the fraction of photosynthetically active radiation (FPAR) absorbed by the vegetation layer; and 0.5 represents the proportion of the effective solar radiation relative to the total solar radiation and with a wavelength of 0.4–0.7 µm that can be utilized by vegetation [33]. FPAR is represented by NDVI and vegetation type, and it does not exceed 0.95 in the equation [34]:

(3) $$FPAR=min\left(\frac{SR\left(x,t\right)-SR_{min}}{SR_{max}-SR_{min}},0.95\right)$$

where *SR*(*x*, *t*) represents the ratio index at pixel *x* in month *t*; *SR_min_* is 1.08, and *SR_max_*, ranging from 4.14 to 6.17, is related to vegetation type. We calculated *SR*(*x*, *t*) using *NDVI*(*x*, *t*) as follows:

(4) $$SR\left(x,t\right)=\frac{1+NDVI\left(x,t\right)}{1-NDVI\left(x,t\right)}$$

The light energy utilization rate (*ε*), subject to temperature and water conditions, refers to the efficiency of the conversion of PAR absorbed by vegetation into organic carbon. We computed it as follows:

(5) $$\varepsilon \left(x,t\right)=T_{\varepsilon 1}\left(x,t\right)\times T_{\varepsilon 2}\left(x,t\right)\times W_{\varepsilon }\left(x,t\right)\times \varepsilon_{max}$$

where *T*_*ε*1_(*x*, *t*) and *T*_*ε*2_(*x*, *t*) represent the effects of temperature on *ε*; *W_ε_* represents the effect of water on *ε*, and *ε_max_* represents the maximum *ε* under ideal conditions (g C/MJ).

Estimation of temperature stress factors: *T*_*ε*1_(*x*, *t*) and *T*_*ε*2_(*x*, *t*) reflect the effects of temperature on *ε*. We calculated the former as follows:

(6) $$T_{\varepsilon 1}\left(x,t\right)=0.8+0.02\times T_{opt}\left(x\right)-0.0005\times {\left[T_{opt}\left(x\right)\right]}^{2}$$

where *T_opt_*(*x*) represents the average temperature (°C) of the month when the NDVI of a certain area reaches the maximum in a year. If the average temperature of a month is less than or equal to −10 °C, *T_opt_*(*x*) is set to 0.

(7) $$T_{\varepsilon 2}\left(x,t\right)=\frac{1.184}{\left\{1+\exp\left[0.2\times \left(T_{opt}\left(x\right)-10-T\left(x,t\right)\right)\right]\right\}}\times \frac{1}{\left\{1+\exp\left[0.3\times \left(-T_{opt}\left(x\right)-10+T\left(x,t\right)\right)\right]\right\}}$$

If *T*(*x*, *t*), the average temperature of a month is 10 °C higher or 13 °C lower than *T_opt_*(*x*); then, the *T*_*ε*2_(*x*, *t*) value for this month is half the value of *T*_*ε*2_(*x*, *t*) for which *T*(*x*, *t*) is *T_opt_*(*x*).

Estimation of water stress factors: *W_ε_*(*x*, *t*) reflects the effect of water on the light utilization efficiency of plants. It gradually increases with the rise in effective water in the environment, and its value ranges from 0.5 (under extreme drought conditions) to 1 (under very wet conditions). We calculated it as follows:

(8) $$W_{\varepsilon }\left(x,t\right)=0.5+0.5\times E\left(x,t\right)/E_{p}\left(x,t\right)$$

where *E*(*x*, *t*) represents the actual evapotranspiration (mm) of a region based on the actual evapotranspiration model of Zhou et al. [10], and *E_p_*(*x*, *t*) represents the potential evapotranspiration of a region based on the complementary relationship proposed by Boucher [35].

Determination of *ε_max_*: We determined the value of *ε_max_* through Zhu’s method of simulating the *ε_max_* of different vegetation types based on the principle of error minimization [36].

#### 2.2.2. Linear Regression Analysis

The annual change rate of NPP based on the total annual NPP of forest vegetation in the same period was calculated through a linear regression model [37,38]. The pixel-by-pixel change rate of NPP was calculated based on the average annual forest NPP for all the pixels from 1982 to 2015, as shown in the equation:

(9) y=αt+β+ε

where *t* is a year in the time series; *α* is the regression coefficient indicating the annual change rate of NPP; *β* is the regression constant; and *ε* is the fitted residual. A value of *p* < 0.05 (or 0.01) indicated that a linear regression coefficient was significantly based on the *t*-test.

#### 2.2.3. Partial Correlation Analysis

The interference of other variables on the impacts of climatic factors on NPP was eliminated through second-order partial correlation analysis [39,40]. The pixel-by-pixel partial correlation coefficient (r) of forest NPP was calculated with total solar radiation, temperature, and precipitation. The partial correlation coefficient between NPP and temperature was calculated while solar radiation and precipitation were fixed, whereas that between NPP and precipitation was determined while solar radiation and temperature were set, and that between NPP and solar radiation was computed while temperature and precipitation were constant [41].

#### 2.2.4. Sliding Partial Correlation Analysis

The sliding correlation coefficient can be used to investigate the variations in the impacts of climatic factors on vegetation NPP [40]. The 10–20a is considered to be the appropriate range of years for a moving window in sliding correlation analysis [42]. Thus, in order to determine the temporal variations in the relationship between vegetation NPP and climate despite the limited study period, we selected 17a as the sliding stride to calculate the second-order partial correlation coefficient of forest NPP with temperature (R_NPP-T_), precipitation (R_NPP-P_), and total solar radiation (R_NPP-S_). A sequence for each correlation coefficient from 1982 to 1998, 1983 to 1999, …, and 1999 to 2015 was obtained. Thus, the variations in the correlation coefficient between the NPP of China’s forest vegetation and the climatic factors with time were analyzed, and statistical significance tests were performed for such changes. Values of *p* < 0.05 indicated that the responses of forest NPP to climatic factors were statistically significant.

## 3. Results

### 3.1. Temporal Variations in Forest NPP

**Figure 1:**
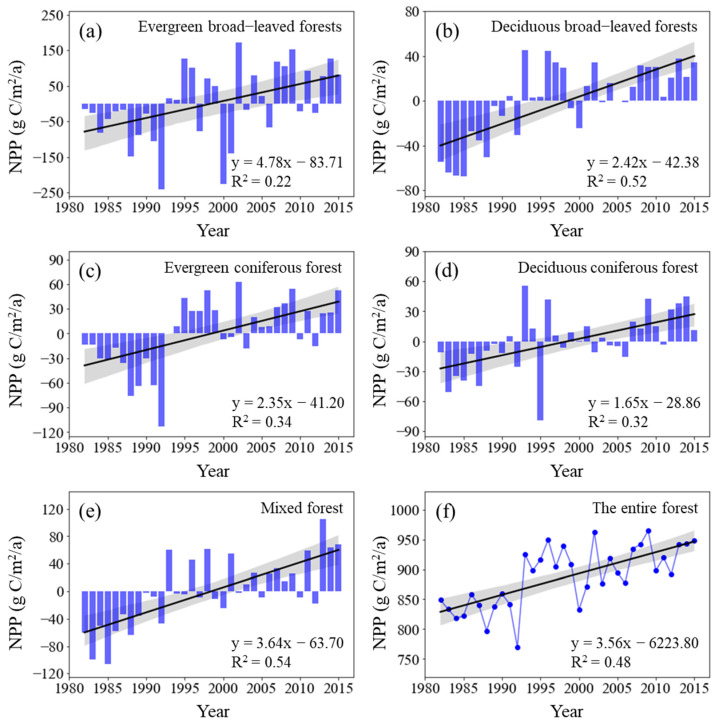
Interannual change in NPP across forest types from 1982 to 2015 (Here, the vertical solid rectangles represent NPP anomalies across forest types; the black lines represent the interannual change trend in forest NPP). Shading denotes 95% prediction intervals.

The total NPP of China’s forest vegetation from 1982 to 2015 was between 770 × 10^12^ g C/a and 965 × 10^12^ g C/a, and the average annual NPP was 887 × 10^12^ g C/a. From the perspective of different vegetation types, the NPP per unit area of evergreen broad-leaved forests was the highest (1323.71 g C/m^2^/a), followed by mixed forests (832.06 g C/m^2^/a) and deciduous broad-leaved forests (637.21 g C/m^2^/a). The NPP per unit area of evergreen coniferous forests and deciduous coniferous forests was 497.59 g C/m^2^/a and 442.35 g C/m^2^/a, respectively. All five types of forests showed a significantly increasing trend in NPP during the study periods (Figure 1). The NPP growth rate of evergreen broad-leaved forests was the highest (4.78 g C/m^2^/a; *p* < 0.01), followed by mixed forests (3.64 g C/m^2^/a; *p* < 0.01), deciduous broad-leaved forests (2.42 g C/m^2^/a; *p* < 0.01), and evergreen coniferous forests (2.35 g C/m^2^/a; *p* < 0.01), whereas that of deciduous coniferous forests was the lowest (1.65 g C/m^2^/a; *p* < 0.01). The total NPP of forest vegetation in China extensively increased by 11.67% from 848.86 × 10^12^ g C in 1982 to 947.89 × 10^12^ g C in 2015 and at a linear rate of 3.558 × 10^12^ g C/a per year (*p* < 0.01) over thirty years (Figure 1).

### 3.2. Spatial Variations in Forest NPP

**Figure 2:**
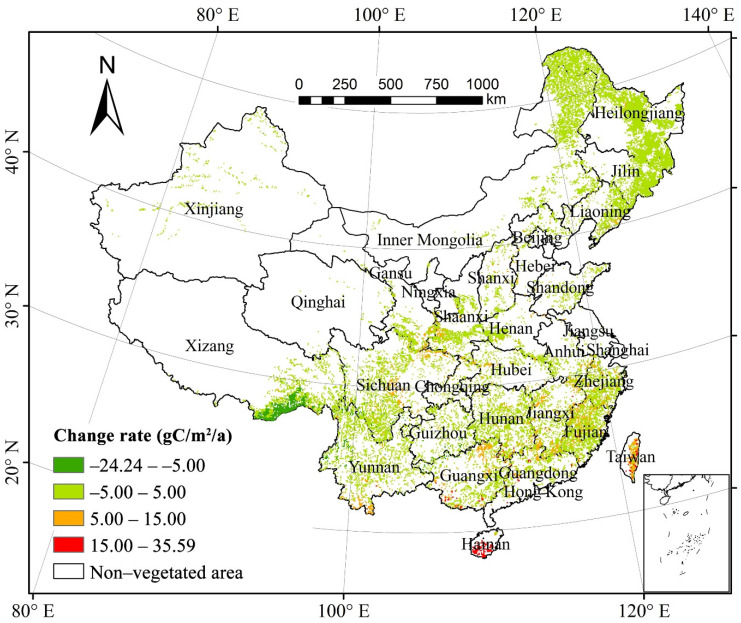
Spatial distribution of China’s forest NPP change from 1982 to 2015.

The annual difference in forest NPP from 1982 to 2015 was between −24.23 g C/m^2^/a and 35.59 g C/m^2^/a, with an average growth rate of 2.36 g C/m^2^/a. The NPP of 89.25% of the total forest area increased, whereas that of only 10.45% decreased. About 51.40% of forest vegetation in the Changbai Mountains, Sichuan–Shaanxi–Gansu bordering region, the Xiaoxinganling Mountains, the northern Daxinganling Mountains, the Han River valley, southern Yunnan, and Eastern Taiwan showed a substantial increase (*p* < 0.05) in their NPP. Only 1.33% of forest vegetation in the Bhareli River and Subansiri River regions in Southeastern Tibet showed a noteworthy decrease (*p* < 0.05) in their NPP. Our results demonstrated that the area of forest vegetation with increasing NPP was greater than that with decreasing NPP and that the NPP of the entire forest increased over thirty years (Figure 2).

Based on the annual difference in NPP per unit area of the forest types, the areas of mixed forests with significant increase (*p* < 0.05) comprised the largest proportion (83.33%) of the total area of mixed forests; those of deciduous broad-leaved forests and deciduous coniferous forests with substantial increase both exceeded 60%; whereas those of evergreen broad-leaved forests and evergreen coniferous forests with considerable increase accounted for 44.06% and 33.89%, respectively. On the other hand, the proportions of all forest vegetation types with a notable decrease (*p* < 0.05) in NPP were low. The area of mixed forests with significant decrease in NPP per unit area was almost zero; all those of deciduous broad-leaved forests, evergreen coniferous forests, and deciduous coniferous forests with substantial decrease in NPP per unit area were less than 1%; whereas those of evergreen broad-leaved forests with noteworthy decrease in NPP per unit area comprised the largest proportion that was only 6.67%. Our findings showed that the area of each forest vegetation type with a significant increase (*p* < 0.05) in NPP per unit of area covered a proportion larger than that with significant decrease (*p* < 0.05), and that the NPP of the five forest types generally increased during the study periods.

### 3.3. Interannual Correlation between Forest NPP and Climatic Factors

#### 3.3.1. Total Annual NPP and Temperature

**Figure 3:**
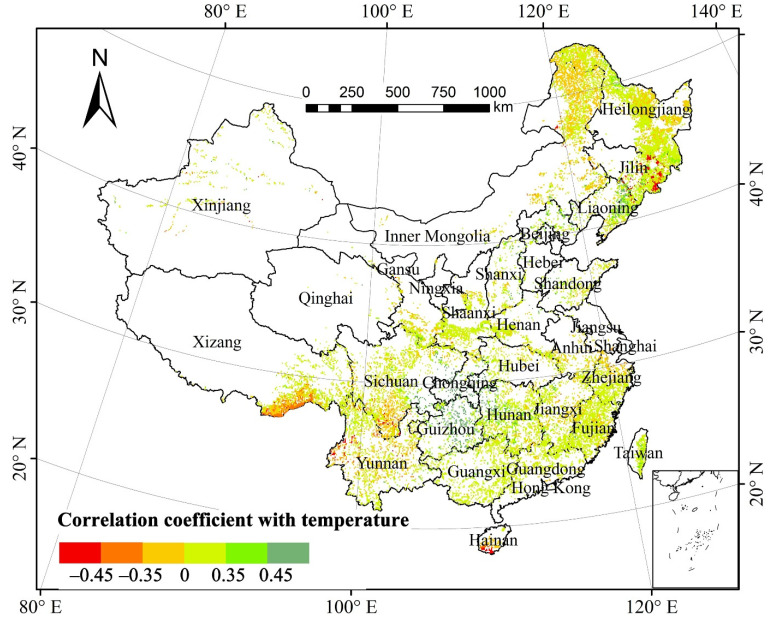
Spatial distribution of partial correlation coefficients between forest NPP and mean annual temperature from 1982 to 2015.

The partial correlation coefficient of the total annual forest NPP and mean annual temperature (MAT) was 0.558 at a 0.01 significant level, suggesting that forest NPP was significantly correlated with MAT in the past three decades (Figure 3). Spatially, the areas with a positive correlation between total forest NPP and MAT accounted for 70.92% of the total forest area (Table 1). Approximately 8.60% of the areas in the northeastern region of the Yunnan–Guizhou Plateau, Chongqing, the southeastern region of Sichuan Basin, and western Hunan passed the significance test (*p* < 0.05). The areas with negative correlation between forest NPP and MAT accounted for 29.08%, and those with significantly negative correlation were mainly distributed in southern Hainan and the Bhareli River as well as in the Subansiri River in Tibet. The areas in southeastern Jilin and southern Hainan that passed the significance test (*p* < 0.05) accounted for only 3.24%.

The pixels of the mixed forests with significant correlation between the NPP of different forest types and MAT (*p* < 0.05) accounted for 36.01%, which is the greatest proportion, of the total pixels of mixed forests; those of green broad-leaved forests (*p* < 0.05) accounted for 20.27%; those of deciduous broad-leaved forests and evergreen coniferous forests (*p* < 0.05) both comprised greater than 10%; whereas those of deciduous broad-leaved forests (*p* < 0.05) accounted for slightly higher than 7%. The pixels of other forest types, except the deciduous broad-leaved forests, with the positive correlation between NPP and MAT, generally constituted proportions greater than those with negative correlation, indicating that temperature evidently altered the NPP across different forest types.

#### 3.3.2. Total Annual NPP and Precipitation

**Figure 4:**
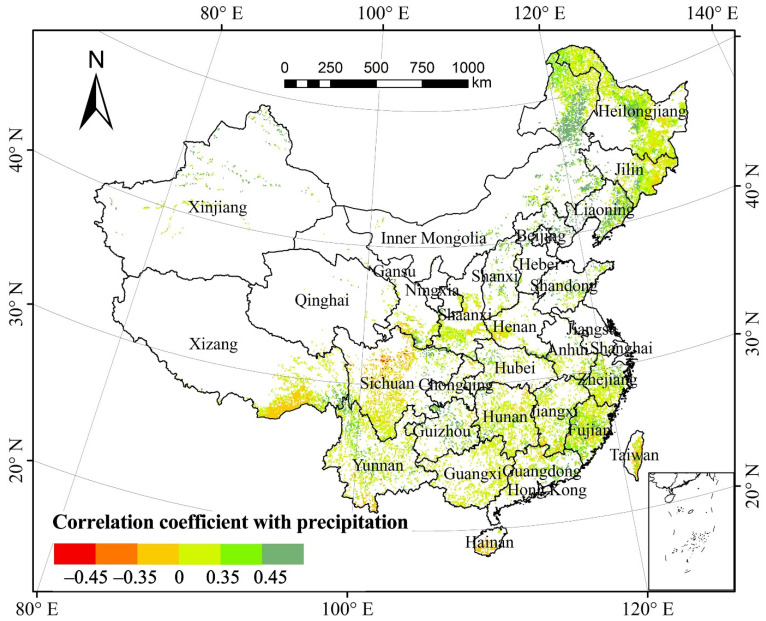
Spatial distribution of partial correlation coefficients between forest NPP and mean annual precipitation from 1982 to 2015.

The partial correlation coefficient of the total annual forest NPP and the mean annual precipitation (MAP) was 0.167 (*p* > 0.05), suggesting that there was no significant correlation between the total annual forest NPP and MAP in the past three decades (Figure 4). Spatially, the areas with a positive correlation between forest NPP and MAP accounted for 88.10% of the total forest area (Table 1). About 31.72% of the areas, mainly in the Tibet–Yunnan–Sichuan bordering regions, eastern and western Liaoning, southeastern Guizhou, the Daxinganling Mountains, southeastern Liaoning, the Xiaoxinganling Mountains, Shandong, the Changbai Mountains, Fujian, Yunnan, eastern Guizhou, southern Jiangxi, and eastern Guangdong showed a significantly positive correlation (*p* < 0.05). The areas with negative correlation between forest NPP and MAP accounted for 11.90%. Only 0.28% of the areas mainly in northern Sichuan passed the significance test (*p* < 0.05).

Based on the proportion of pixels of forest type with correlation between NPP and MAP relative to the total pixels of the same vegetation type, the NPP of 49.47% of deciduous coniferous forests, 38.54% of deciduous broad-leaved forests, 32.96% of mixed forests, 25.19% of evergreen coniferous forests, and 12.66% of evergreen broad-leaved forests were significantly affected by precipitation (*p* < 0.05). All the areas in the five forest vegetation types with a positive correlation between NPP and precipitation comprised a proportion greater than those with a negative correlation. Hence, precipitation minimally affected the forest NPP in the study periods.

#### 3.3.3. Total Annual NPP and Total Solar Radiation

**Figure 5:**
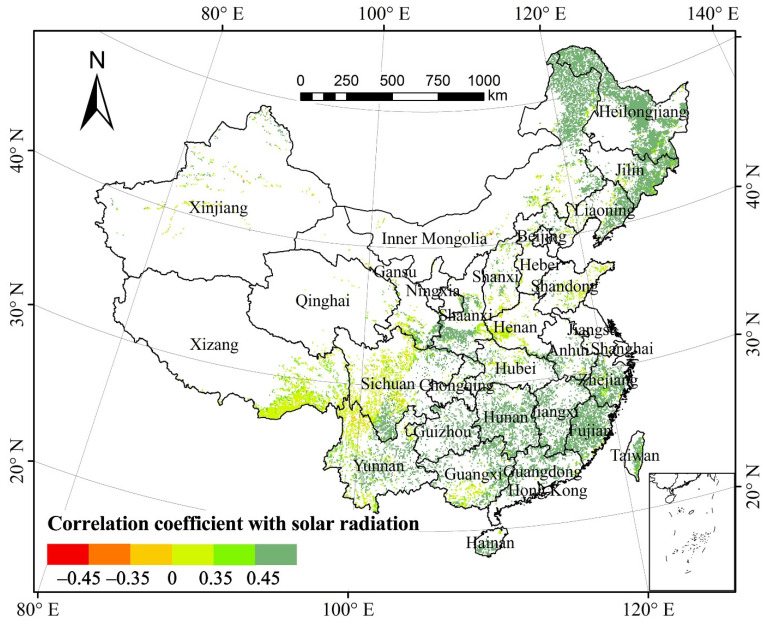
Spatial distribution of partial correlation coefficients between forest NPP and total solar radiation from 1982 to 2015.

The partial correlation coefficient of the total annual NPP of forest vegetation and total solar radiation (TSR) was 0.476 at 0.05 significant level, suggesting that the total annual forest NPP was significantly correlated with TSR in the past three decades (Figure 5). Spatially, the areas with a positive correlation between forest NPP and TSR accounted for 98.89% of the total forest area (Table 1). About 80.22% of the areas, mainly in the Daxinganling Mountains, eastern Liaoning, Han River valley, Xiaoxinganling Mountains, and south of the Yangtze River, passed the significance test (*p* < 0.05). The areas with the negative correlation between forest NPP and TSR accounted for 1.11%. Those mainly in Sichuan, northwestern Yunnan, and Xinjiang that passed the significance test (*p* < 0.05) only accounted for 0.03% of the total forest area.

Based on the proportion of pixels of each forest type showing correlation between NPP and TSR relative to the total pixels of the corresponding forest type, the proportion of areas across forest types with significantly positive correlation (*p* < 0.05) exceeded 60%, and those with significantly negative correlation (*p* < 0.05) were almost zero. Thus, the increase in solar radiation notably facilitated the growth of forest NPP in the study periods.

### 3.4. Varying Responses of Forest NPP to Climate Change

#### 3.4.1. Dynamic Relationship between Forest NPP and Temperature

**Figure 6:**
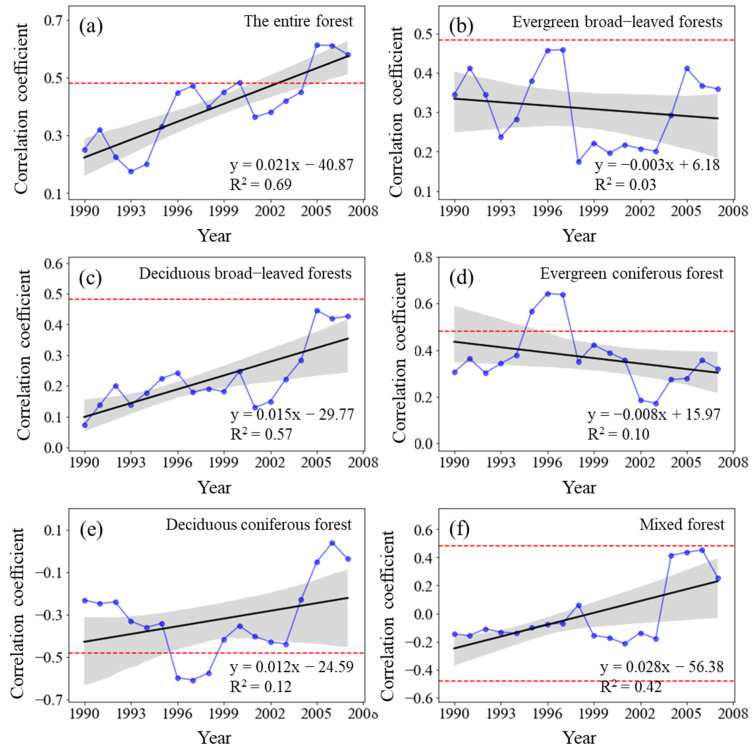
Variations in the sliding correlation coefficients between NPP and mean annual temperature across forest types. Statistically significant partial correlation coefficients are indicated as dotted lines (*p* < 0.05). The black lines represent the interannual change trend in partial correlation coefficients. Shading denotes 95% prediction intervals.

The sliding correlation coefficient R_NPP-T_ between the NPP of the entire forest from 1982 to 2015 and MAT significantly increased (*p* < 0.01) (Figure 6). Similarly, the R_NPP-T_ for deciduous broad-leaved forests and mixed forests also considerably increased (*p* < 0.01). Although the R_NPP-T_ for deciduous coniferous forests increased, it failed the significance test (*p* > 0.05). The R_NPP-T_ for evergreen broad-leaved forests and evergreen deciduous forests decreased, and it failed the significance test (*p* > 0.05).

Considering different time periods, the positive correlation between forest NPP and MAT gradually weakened from the 1982–1998 period to the 1985–2001 period, but it steadily strengthened after 1986–2002 and slowly declined again after 1993–2009 until 1999–2015. The deciduous broad-leaved forests showed an increasingly positive correlation between NPP and MAT, whereas the evergreen coniferous forests and evergreen broad-leaved forests demonstrated no significant change in their corresponding positive correlation. The pertinent correlation in the deciduous coniferous forests and mixed forests changed from negative to positive and continuously increased.

#### 3.4.2. Dynamic Relationship between Forest NPP and Precipitation

**Figure 7:**
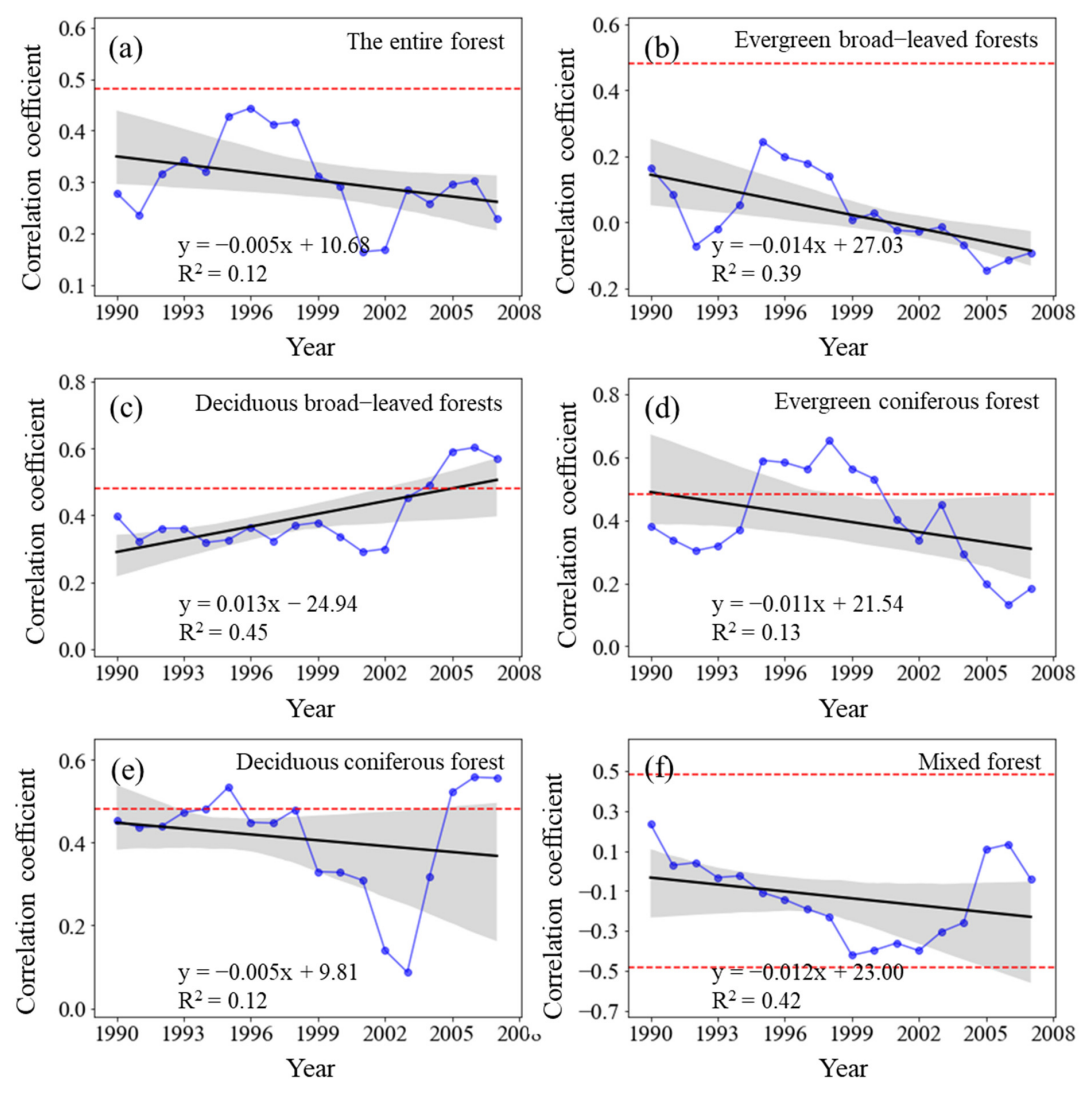
Variations in the sliding correlation coefficients between NPP and mean annual precipitation across forest types. Statistically significant partial correlation coefficients are indicated as dotted lines (*p* < 0.05). The black lines represent the interannual change trend in partial correlation coefficients. Shading denotes 95% prediction intervals.

The sliding correlation coefficient R_NPP-P_ between the NPP of the entire forest from 1982 to 2015 and MAP showed a fluctuating downward trend (*p* > 0.05) (Figure 7). The R_NPP-P_ of evergreen broad-leaved forests significantly decreased (*p* < 0.01), whereas that of deciduous broad-leaved forests substantially increased (*p* < 0.01). The R_NPP-P_ of evergreen coniferous forests increased (*p* > 0.05), whereas that of deciduous coniferous forests and mixed forests decreased (*p* > 0.05).

The R_NPP-P_ in all the periods showed positive correlation and failed the significance test of *p* < 0.05, but the underlying change was periodic. The R_NPP-P_ increased from the 1982–1998 period to the 1993–2009 period, gradually decreased from the 1988–2004 period to the 1996–2012 period, and steadily increased again after 1994–2010, but it did not reach a significantly positive correlation. The R_NPP-P_ of evergreen broad-leaved forests changed from positive to negative, but it was not statistically significant, whereas that of deciduous broad-leaved forests and evergreen coniferous forests was positive in all the study periods. Nevertheless, the changes varied as the R_NPP-P_ of deciduous broad-leaved forests increased annually, whereas that of evergreen coniferous forests decreased yearly. The R_NPP-P_ of deciduous coniferous forests similarly changed, and that of the mixed forests changed from positive to negative.

#### 3.4.3. Dynamic Relationship between Forest NPP and Solar Radiation

**Figure 8:**
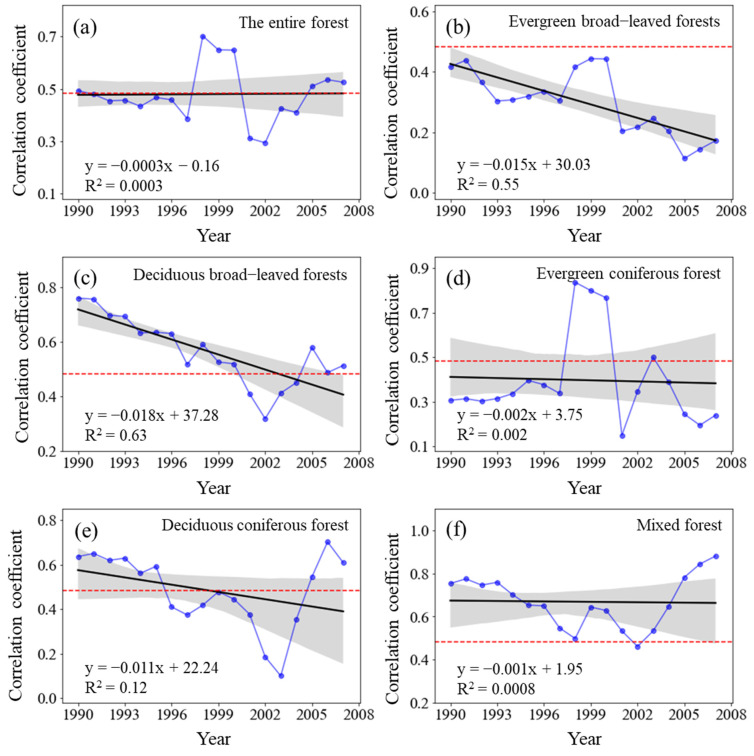
Variations in the sliding correlation coefficients between NPP and total solar radiation across forest types. Statistically significant partial correlation coefficients are indicated as dotted lines (*p* < 0.05). The black lines represent the interannual change trend in partial correlation coefficients. Shading denotes 95% prediction intervals.

The sliding correlation coefficient R_NPP-S_ between the NPP of the entire forest from 1982 to 2015 and TSR did not significantly change (*p* > 0.05) (Figure 8) and the R_NPP-S_ was positive in each sliding window. The correlation between forest NPP and TSR was significantly positive from 1982 to 1998 (*p* < 0.05), but it gradually weakened from the 1983–1999 period to 1989–2005 period. Its correlation coefficient reached a significantly positive correlation (*p* < 0.05) from the 1990–2006 period to the 1992–2008 period. It sharply declined but remained positively correlated from 1993 to 2009, after which it gradually increased but did not show a significantly positive correlation (*p* < 0.05) until 1998–2014. However, the notable decrease in the R_NPP-S_ of all the forest types demonstrated the dynamic responses of forest NPP to TSR. The R_NPP-S_ of evergreen broad-leaved forests and deciduous broad-leaved forests significantly decreased (*p* < 0.01).

## 4. Discussion

In this study, we simulated China’s forest NPP and investigated the spatiotemporal patterns of forest NPP from 1982 to 2015. Even though the CASA model has been widely applied to NPP in China [1,32], the calculation still needs to be verified. Thus, we compared the NPP calculated from the CASA model with other simulation results in the literature. The mean annual NPP estimated for the entire forest (887 × 10^12^ g C/a) in this study was similar to the forest biomass given by Zhan et al. [43] (approximately 840.3 × 10^12^ g C/a) and Ni [12] (738.9 × 10^12^ g C/a). However, it was about twice the estimate of Fang et al. [11] (461.0 × 10^12^ g C/a) between 1949 and 1998. The average NPP per unit area estimated for the entire forest in this study was slightly lower than that of the MODIS MOD17A3 products (666.19 g C/m^2^/a) with a spatial resolution of 1000 m at an annual interval (https://modis.gsfc.nasa.gov/ (accessed on 9 October 2022)), which is commonly used to explore the spatiotemporal patterns of NPP on regional and global scales. Moreover, our estimate of the NPP per unit area of evergreen broad-leaved forests was close to that reported by Wu et al. [44] (1327.22 g C/m^2^/a); that of deciduous broad-leaved forests was consistent with those provided by Ni [12] (671.80 g C/m^2^/a), Liang et al. [13] (688.50 g C/m^2^/a), and Zhu et al. [36] (642.90 g C/m^2^/a); that of evergreen coniferous forests was similar to those determined by Wu et al. [44] (515.69 g C/m^2^/a), Jiang et al. [45] (519.34 g C/m^2^/a), and Liang et al. [13] (542.80 g C/m^2^/a); that of deciduous coniferous forests was similar to those determined by Zhu et al. [36] (438.80 g C/m^2^/a) and Liang et al. [13] (401.40 g C/m^2^/a); and that of the mixed forests was consistent with those reported by MOD17A3 (749.65 g C/m^2^/a). Despite the closeness of our pertinent estimates with those in the literature, there were still differences, and they were possible owing to the discrepancies in research periods, data sources, study areas, vegetation types, and classification accuracy. In addition, more detailed field measurements on NPP should be examined in our future studies in order to accurately explore the spatiotemporal pattern of forest NPP in China.

From the spatial perspective, the forest NPP in this study gradually decreased from southeast to northwest, consistent with the spatial distribution of precipitation in China [1,13]. Furthermore, we found that forest NPP was mainly distributed in the humid or very humid monsoon regions of China. The spatial pattern of NPP is generally in line with previous studies [32,46]. For instance, the areas with forest NPP ranging from 1000 to 2100 g C/m^2^/a were distributed in Eastern Taiwan, southwestern Yunnan, and southern Hainan, where the main vegetation type is evergreen broad-leaved forests and where productivity was generally high due to abundant precipitation, warm climate, and rich groundwater. Contrarily, annual NPP below 200 g C/m^2^/a was mainly in the Qinghai–Tibetan Plateau and Xinjiang Regions, where the climate is characterized by a low precipitation amount. In addition, our findings of the annual NPP at the biome level also agreed well with previous studies [13,46]. For example, evergreen forests exhibited a higher NPP than deciduous forests. The highest NPP witnessed in the evergreen broad-leaved forest, with an average range of 1000–2100 g C/m^2^/a. The deciduous coniferous forest has the lowest annual NPP (mostly 200–600 g C/m^2^/a) in areas with either dry climate or low temperature.

On average, forest NPP in China significantly increased during the past three decades. Despite its overall trend as a whole, NPP trends in the forest showed an obvious geographical heterogeneity. The overall rising trends in NPP are also consistent among biomes, with the increasing trends being all statistically significant at a significant level of 0.01. Such upward trends in forest NPP were consistent with the increasing NPP of terrestrial vegetation in the northern hemisphere [13], indicating that forest vegetation in China played an increasingly irreplaceable role in carbon sequestration in the past few decades [9,13].

Relative to other areas, those covered with forest vegetation had a significant increase in mean annual temperature and total annual solar radiation but had a decrease in annual precipitation from 1982 to 2015. The annual increase in temperature improved the forest NPP of these areas because it may have prolonged the growth period of their forest vegetation [47] and controlled on plant metabolic activity, water and nutrient availability [48]. The yearly increase in solar radiation also augmented the NPP of the forested areas probably because it increased the rate of photosynthesis among the flora, facilitated the accumulation of organic matter, and intensified the warmth therein. Conversely, the decline in precipitation diminished the forest NPP because it may have reduced the water supply essential for the growth of the forest vegetation. By analyzing the relationship between climatic factors and NPP, we found precipitation had a lower correlation with NPP than temperature and solar radiation, implying that the effect of annual precipitation on forest vegetation growth was smaller than other climatic factors, which was also presented by the previous study on biogeographic patterns of China’s forests growth rate and their climatic control [6]. Our findings even showed that forest NPP significantly increased only in eastern Taiwan, southern Yunnan, and south of the Yangtze River, where southeast and southwest monsoon systems were dominant and where rainfall from the Pacific Ocean and the Indian Ocean was abundant during the growing season. As a whole, we showed that ongoing climate change could exert positive impacts on forest growth and carbon sequestration capacity, which was widely acknowledged by previous studies [1,8,9].

Previous researchers have also investigated the climate-driven effect on variations in forests NPP [6,13,22]. However, little is known about the temporal differentiation in the relationships between forests NPP and climate variables, which is essential for future forest ecology and management. Our results of the sliding partial correlation analysis in each 17-year moving window indicated that the interannual partial correlation coefficient between annual NPP and annual TSR (R_NPP-S_) showed little change, that between annual NPP and MAT (R_NPP-T_) significantly increased, but that between annual NPP and MAP (R_NPP-P_) displayed a fluctuating downward trend. Obviously, the correlations exhibited temporally varying strength in our study, which also has been reported by previous studies [29,40,49]. However, unlike previous studies [25,50,51], we did not detect a loss of sensitivity of forest vegetation growth to increasing warming. Instead, we find that the impacts of precipitation on forest NPP were also weakening as the annual precipitation amount decreased, which was similar to the earlier findings that the relationship between NPP and MAP varied with the rising in MAP [52]. Given that the mechanistic explanation for varying forest response to environmental change was still unknown, a complete understanding of the combined environmental effect on forest ecosystem productivity remained a great challenge for further studies.

## 5. Conclusions

Overall, the interannual NPP of the entire forest and its corresponding vegetation types in China from 1982 to 2015 significantly increased. Our findings demonstrated that climatic factors could variably affect the NPP of forests, as the increase in temperature and solar radiation enhanced the interannual forest NPP, whereas the decrease in precipitation reduced the forest NPP. However, such responses of the forest NPP to climatic factors constantly changed, and they varied with the type of forest vegetation. Studies on the dynamics of the correlation between forest NPP and climatic factors are essential for elucidating the adaptability of forests to global warming.

## Figures and Tables

**Table 1:** Percentages of the area with significant partial correlations (*p* < 0.05) between forest NPP and climatic factors. The numbers in parentheses indicate the percentage of area with significant partial correlations between forest NPP and climatic factors.

Climatic Factors	Positive	Negative
Temperature	70.92% (8.60%)	29.08% (3.24%)
Precipitation	88.10% (31.72%)	11.90% (0.28%)
Solar radiation	98.89% (80.22%)	1.11% (0.03%)

## Data Availability

The data used in the present work have been listed in the Data Sources.

## Supplementary Materials

The following supporting information can be downloaded at: https://www.mdpi.com/article/10.3390/plants11212932/s1, Figure S1: China’s forest distribution and meteorological stations.
