# Supplementary material for: Responses of Forest Net Primary Productivity to Climatic Factors in China during 1982–2015
Source: Plants (Basel). 2022 Oct 31;11(21):2932. doi: 10.3390/plants11212932 (PMC9656275; doi:10.3390/plants11212932)
Supplement: Supplementary file 1 [file plants-1989403-supplementary.pdf › plants-1989403-supplementary.pdf]

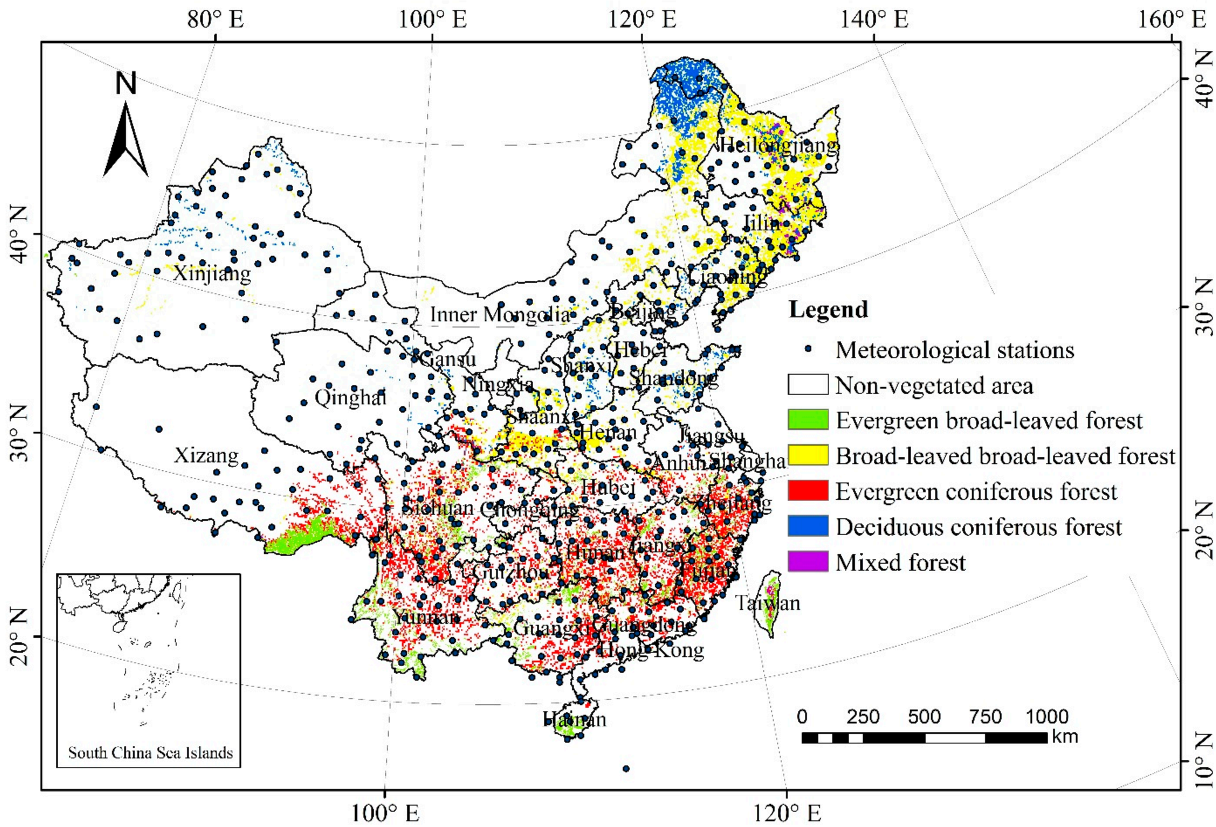

Figure S1 China's forest distribution and meteorological stations
